# Analysis of the human *Alu *Ye lineage

**DOI:** 10.1186/1471-2148-5-18

**Published:** 2005-02-22

**Authors:** Abdel-Halim Salem, David A Ray, Dale J Hedges, Jerzy Jurka, Mark A Batzer

**Affiliations:** 1 Department of Biological Sciences, Biological Computation and Visualization Center, Center for Bio-Modular Multi-scale Systems, Louisiana State University, 202 Life Sciences Building, Baton Rouge, Louisiana 70803 USA; 2 Department of Anatomy, Suez Canal University, Ismailia, Egypt; 3 Genetic Information Research Institute, 2081Landings Drive, Mountain View, CA 94043 USA

## Abstract

**Background:**

*Alu *elements are short (~300 bp) interspersed elements that amplify in primate genomes through a process termed retroposition. The expansion of these elements has had a significant impact on the structure and function of primate genomes. Approximately 10 % of the mass of the human genome is comprised of *Alu *elements, making them the most abundant short interspersed element (SINE) in our genome. The majority of *Alu *amplification occurred early in primate evolution, and the current rate of Alu retroposition is at least 100 fold slower than the peak of amplification that occurred 30–50 million years ago. *Alu *elements are therefore a rich source of inter- and intra-species primate genomic variation.

**Results:**

A total of 153 *Alu *elements from the Ye subfamily were extracted from the draft sequence of the human genome. Analysis of these elements resulted in the discovery of two new *Alu *subfamilies, Ye4 and Ye6, complementing the previously described Ye5 subfamily. DNA sequence analysis of each of the *Alu *Ye subfamilies yielded average age estimates of ~14, ~13 and ~9.5 million years old for the *Alu *Ye4, Ye5 and Ye6 subfamilies, respectively. In addition, 120 *Alu *Ye4, Ye5 and Ye6 loci were screened using polymerase chain reaction (PCR) assays to determine their phylogenetic origin and levels of human genomic diversity.

**Conclusion:**

The *Alu *Ye lineage appears to have started amplifying relatively early in primate evolution and continued propagating at a low level as many of its members are found in a variety of hominoid (humans, greater and lesser ape) genomes. Detailed sequence analysis of several *Alu *pre-integration sites indicated that multiple types of events had occurred, including gene conversions, near-parallel independent insertions of different *Alu *elements and *Alu*-mediated genomic deletions. A potential hotspot for *Alu *insertion in the Fer1L3 gene on chromosome 10 was also identified.

## Background

The proliferation of *Alu *elements has had a significant impact on the architecture of primate genomes [1]. They comprise over 10% of the human genome by mass and are the most abundant short interspersed element (SINE) in primate genomes [2]. *Alu *elements have achieved this copy number by duplicating via an RNA intermediate in a process termed retroposition [3]. During retroposition the RNA copy is reverse transcribed by target primed reverse transcription (TPRT) and subsequently integrated into the genome [4-6]. While unable to retropose autonomously, *Alu *elements are thought to borrow the factors that are required for their amplification from the LINE (long interspersed element) elements [6-9], which encode a protein with endonuclease and reverse transcriptase activity [10,11]. Because of their high copy number, *Alu *repeats have been a significant source of new mutations as a result of insertion and post-integration recombination between elements [12,13].

The majority of *Alu *amplification occurred early in primate evolution, and the current rate of *Alu *retroposition is at least 100 fold slower than the peak of amplification that appears to have occurred 30–50 million years ago [2,14-16]. Even though there are over one million *Alu *elements within the human genome, only a small number of these elements are capable of movement [17]. As a result of the limited amplification capacity of *Alu *elements, a series of discrete subfamilies of *Alu *elements that share common diagnostic mutations have been identified in the human genome [18-21]. A small subset of "young" *Alu *repeats are so recent in origin that they are present in the human genome and absent from the genomes of non-human primates, with some of the elements being polymorphic with respect to insertion presence/absence in diverse human genomes [16,22-25]. Individual SINE elements have proven to be essentially homoplasy-free characters which are therefore quite useful for resolving phylogenetic and population genetic questions [2,26-34]. For example, young *Alu *subfamilies which arose around the radiation of Subtribe Hominina (gorillas, chimpanzees, and humans) four to six million years ago [35] were used as homoplasy free phylogenetic markers to resolve the branching order in hominids [36]. Relationships among other primates have also been resolved using relatively large numbers of *Alu *elements as phylogenetic markers [28,37-40]

We have previously characterized a large number of recently integrated *Alu *elements found in the human genome that fall in six distinct lineages, termed Ya, Yb and Yc, Yd, Yg and Yi based upon their diagnostic mutations [41-52]. Here, we describe the distribution in the human genome of three *Alu *subfamilies that are members of the *Alu *Ye lineage [53] and are characterized by four (Ye4), five (Ye5) and six (Ye6) diagnostic mutations, respectively.

## Results

### Subfamily size and age

**Figure 1:**
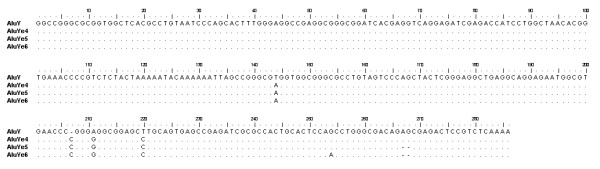
**Sequence alignment of *Alu *Ye subfamilies**. The consensus sequence for the *Alu *Y subfamily is shown at the top. The sequences of *Alu *Ye4, Ye5 and Ye6 subfamilies are shown below. The dots below represent the same nucleotides as the consensus sequence. Deletions are shown as dashes and mutations are shown as the correct base for each of the subfamilies.

*Alu *Ye elements were identified in the draft sequence of the human genome using BLAST [54] queries of the draft sequence to identify exact complements to an *Alu *Ye specific oligonucleotide (Fig. 1). See the Materials and Methods section for details on the search. Using this approach we identified 25 Ye4 subfamily members that shared four diagnostic base positions and thus comprised the *Alu *Ye4 subfamily. We also identified 103 elements that shared five diagnostic base positions and comprise the *Alu *Ye5 subfamily and 25 Ye6 subfamily members that shared six diagnostic base positions and comprised the *Alu *Ye6 subfamily. Each of the subfamilies was named in accordance with standard nomenclature for new *Alu *subfamilies [55].

To estimate the copy number of the Ye4, Ye5 and Ye6 *Alu *subfamilies, we preformed BLAST searches of the draft sequence of the human genome using an *Alu *Ye lineage-specific oligonucleotide to query the database (as outlined in the methods). Seventeen of the 25 Alu Ye4 elements were unique (non-paralogous). There were also 76 unique Ye5 *Alu *elements and 23 unique Ye6 *Alu *subfamily members. Multiple alignments of the *Alu *elements from each subfamily were constructed and the number of mutations from the consensus sequence for each *Alu *subfamily was determined. In each case the mutations were divided into those that occur at CpG dinucleotides and those that occur at non-CpG positions without including small insertions or deletions as described previously [47-49]. The mutations are divided into these two different classes to estimate the average age of each subfamily because the CpG base positions in repeated sequences mutate at a rate that is about six times higher than non-CpG positions [56] as a result of the spontaneous deamination of 5-methylcytosine residues [57].

Mutation densities were calculated for each *Alu *Ye subfamily. For 17 elements from the *Alu *Ye4 subfamily, the non-CpG and CpG mutation densities were 2.1% (83/3944) and 12.5 % (106/850). Using a neutral rate of evolution of 0.15% per million years for non-CpG positions [58] and 0.9% per million years for the CpG base positions [56] along with the average mutation density yields age estimates of 14.03 and 13.86 million years old for the Ye4 subfamily. For the *Alu *Ye5 subfamily 76 elements were analyzed that contained a total of 17632 non-CpG nucleotides and 3800 CpG nucleotides that contained 351 non-CpG and 431 CpG mutations. The mutation densities of the Ye5 subfamily were 1.99% and 11.34% for the non-CpG and CpG nucleotides yielding age estimates based on the average mutation density of 13.27 and 12.60 million years old. For the *Alu *Ye6 subfamily 23 elements were analyzed that contained a total of 5336 non-CpG nucleotides and 1150 CpG nucleotides that contained 86 non-CpG and 92 CpG mutations. The mutation densities of the Ye6 subfamily were 1.61% and 8% for the non-CpG and CpG nucleotides yielding age estimates based on the average mutation density of 10.75 and 8.89 million years old.

### Evolutionary analysis

In order to determine the approximate time of insertion for each *Alu *Ye4, Ye5 and Ye6 subfamily member, we performed a series of PCR reactions using human and non-human primate DNA samples as templates. Unfortunately, not all of the loci identified in the draft sequence were amenable to PCR analysis, as some of them had inserted into other repetitive regions of the genome making the design of flanking unique sequence PCR primers difficult.

For the Ye subfamilies, 120 of the 153 elements identified in the draft human genomic sequence were amplified by PCR. Examination of the orthologous regions of the various species genomes displayed a series of different PCR patterns indicative of the time of retroposition of each of the elements into the primate genomes. Results from a series of these experiments showed a gradient of Ye *Alu *repeats beginning with some elements that are recent in origin and unique to the human genome (e.g. Ye5AH110) and ending with elements that are found within all ape genomes (e.g. Ye5AH148). The distribution of all the Ye elements in various primate genomes is summarized in Additional File 2.

### Gene conversion

Gene conversion between *Alu *elements and in other regions of the human genome exerts a significant influence on the accumulation of single nucleotide diversity within the human genome [2,50]. To estimate the frequency of gene conversion in the *Alu *Ye subfamily members, we compared the sequences of the elements found in the human genome to the consensus sequences of other *Alu *subfamilies. Using this approach, we identified two *Alu *Ye5 subfamily members that appeared to have been subjected to partial gene conversion at their 3' ends. *Alu *Ye5AH70 contains three mutations that are diagnostic for the Yb8/9 subfamily. Similarly, *Alu *Ye5AH173 contains three *Alu *Sc mutations. Each of the sequence exchanges occurred in a short contiguous sequence suggesting that they were products of gene conversion rather than homoplasic point mutations.

We identified one *Alu*-containing locus that was involved in full gene conversion/ replacement event, (Ye5AH181). In this case, the orthologous *Alu *elements have similar flanking sequences and direct repeats, although they are not precisely identical due to the random mutations that accumulated over time. DNA sequence analysis of this locus showed that the *Alu *element of selected new world monkey genomes (spider monkey, woolly monkey and tamarin) belonged to the *Alu *Sg subfamily. This suggests that a gene conversion of an older, pre-existing *Alu *Sg may have introduced the Ye5 sequence in the common ancestor of humans, chimpanzees, gorillas and orangutans. Amplification of this locus was unsuccessful in the old world monkey taxa tested.

### *Alu*-mediated genomic deletions

Two deletions of part of the human genome appeared to be associated with newly inserted *Alu *Ye elements. These deletions were identified at loci Ye5AH24 and Ye5AH27. In the case of Ye5AH24, the deletion was associated with a gene conversion of an *Alu *Y in both orangutan and siamang to AluYe5 in human, bonobo, common chimpanzee and gorilla and involved the removal of about 500 bp from the 3' flanking region. For *Alu *Ye5AH27, the deletion was associated with a gene conversion of an *Alu *Sx element (orangutan and siamang) to AluYe5 (human, bonobo, common chimpanzee and gorilla) and involved the removal of 142 bp from the 3' flanking region. Based on this data, we estimate the frequency of *Alu *retroposition mediated deletions of approximately 1.67% (2/120).

The pre-integration sites for three elements (Ye5AH11, Ye5AH40 and Ye5AH173) did not amplify in any non-human primate species. Previously, the insertion of L1 elements has been shown to be associated with large genomic deletions [59]. Thus, one possible explanation for the absence of pre-integration PCR products would be that a large deletion (>1 kb) occurred at each of these loci during *Alu *integration. If a deletion occurred during the integration of an *Alu *element in the human genome, then the pre-integration product size calculated computationally would be an underestimate of the true size of the locus. To investigate this possibility, we utilized long template PCR reactions of these loci that would facilitate the amplification of larger (up to 25 kb) products. Unfortunately, PCR amplicons were not generated by any of these loci, suggesting that the retrotransposition of these *Alu *elements in humans may have generated deletions greater than 25 kb in size. Alternately, the orthologous loci in non-human primate genomes may have undergone additional mutations at the oligonucleotide primer sites, preventing PCR amplification.

### Independent *Alu *insertions

**Figure 2:**
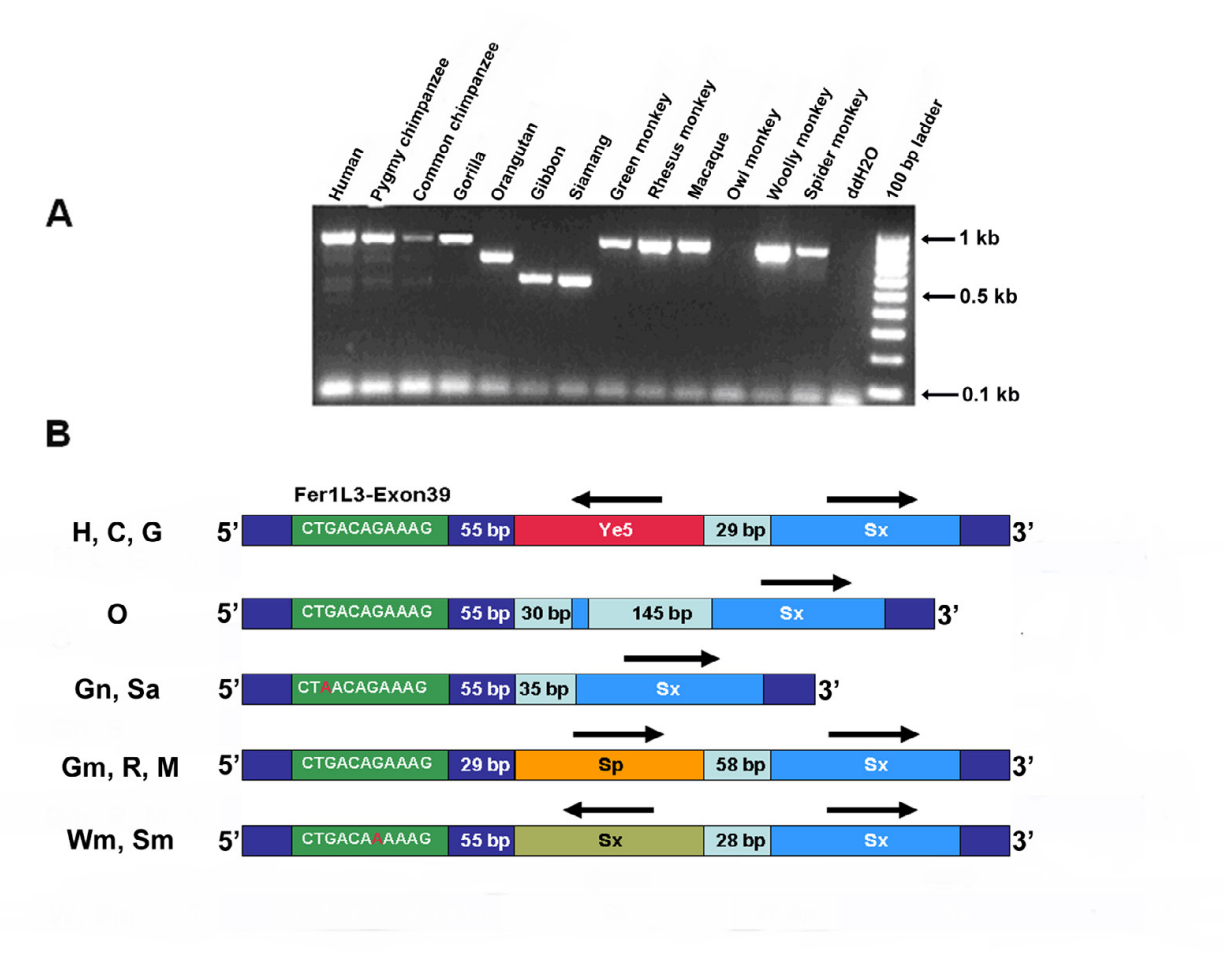
**Parallel insertions at the Ye5AH161 locus. A) **The figure shows an agarose gel chromatograph of the PCR products resulting from amplification at the Ye5AH161 locus in 13 primate species. The ~795 bp PCR product is found in the human, common chimpanzee, pygmy chimpanzee, gorilla, green monkey, Rhesus monkey, macaque, woolly monkey and spider monkey genomes. Smaller bands were found in orangutan, gibbon and siamang. Sequence analysis of the PCR products shows three independent insertions; a Ye5 in subtribe Hominina (human, chimpanzee and gorilla), a second insertion of an *Alu *Sp in old world monkeys, and an *Alu *Sx insertion in new world monkeys. Suspected non-homologous recombination has inserted 145 bp in the orangutan genome at this locus. **B) **A schematic representation of the multiple *Alu *independent insertions and the distance between the shared *Alu *Sx and the independently inserted *Alu *elements. The sequence of Fer1L3-Exon 39 is shown. Silent mutations are highlighted and the distance from the inserted Alus are indicated. Abbreviations used in the figure are: Human (H), Chimpanzee (C), Gorilla (G), Orangutan (O), Gibbon (Gn), Siamang (S), Green monkey (Gm), Rhesus monkey (R), Macaque (M), Woolly monkey (W) and Spider monkey (Sm).

We have also identified one locus (Ye5AH161) that contained multiple paralogous *Alu *insertions in human, chimpanzee, gorilla lineage, old world monkey and new world monkey lineages (Fig. 2). In the human, chimpanzee and gorilla lineage (subtribe Hominina) there was an independent insertion of an *Alu *Ye5 in the 5' flank of an *Alu *Sx that is common to all taxa. In all the old world monkey genomes tested (Green monkey, Macaque and Rhesus monkey), an *Alu *Sp has inserted in the 5' flank of the shared Sx element about 58 bp away of the *Alu *Ye5 present in Hominina. Also, in the woolly and spider monkeys (new world monkeys), there was an independent insertion of an *Alu *Sx in the 5' flank of the shared *Alu *Sx. In gibbon, siamang and orangutan, there were no independent *Alu *insertions at this locus, only the common *Alu *Sx is present. In orangutan, however, there was an extra 145 bp of genomic sequences inserted inside the old *Alu *Sx. The pattern discussed suggests that these three independent parallel insertion events occurred sometime after the divergence of these primates from one another. This locus on chromosome 10q23.33 lies in intron 39–40 of the Human Fer1L3 gene, about 50 bp from exon 39. This locus may be considered a hot spot for *Alu *insertion. An alignment of locus Ye5AH161 is available as Additional file 1 and at .

We also identified another near-parallel independent *Alu *insertion event at human Ye5AH16 locus in all the old world monkey genomes tested (Green monkey, Macaque and Rhesus), within the same locus where an *Alu *Ye5 element was located in the human, chimpanzee, gorilla and orangutan genomes. Thus, the near-parallel insertion most likely occurred after the divergence of humans and apes from old world monkeys, but before the radiation of the old world monkeys. The element present in the old world monkey genomes is an *Alu *Y and is 80 bp from the human insertion site.

### Human genomic diversity

To determine the human genomic diversity associated with each of the *Alu *Ye4, Ye5 and Ye6 subfamily members, we performed a series of PCR reactions on a collection of 80 geographically-diverse human genomes. Using this approach, we identified one new *Alu *insertion polymorphism (Ye5AH167) from the loci analyzed in this report. The allele frequencies, genotypes and heterozygosities for the *Alu *insertion polymorphism are shown in Table 1.

**Table 1:** Human genetic diversity of Ye5AD167.

	**Genotypes**				
Ye5AD167	**+/+**	**+/-**	**-/-**	***f*Ye5**	**Het^1^**

**African American**	6	8	6	0.50	0.51
**Asian**	2	16	2	0.50	0.51
**European/German Caucasian**	3	9	7	0.39	0.49
**South American**	5	13	1	0.61	0.49
**Average Heterozygosity^2^**					0.50

1. Unbiased heterozygosity.

2. The average heterozygosity for all populations.

## Discussion

Our detailed analysis of the *Alu *Ye5 subfamily resulted in the recovery of two new *Alu *subfamilies, Ye4 and Ye6. Each of these *Alu *subfamilies has a relatively small copy number in the human genome. The proportion of polymorphic elements within each of the subfamilies is quite low with only 0.83% of the *Alu *Ye elements being polymorphic, only one member of Ye subfamilies (Ye5AD167) is polymorphic with respect to insertion presence/absence in the human genome. In contrast, many other young *Alu *subfamilies have levels of insertion polymorphism in excess of 20% [2]. Therefore, the amplification of these *Alu *subfamilies within the human genome has occurred at a very low rate, and may have recently ceased entirely. The estimated average ages of ~14, ~13 and ~9.5 million years old for the *Alu *Ye4, Ye5 and Ye6 subfamilies, respectively are consistent with their relatively recent origin in primate genomes. It is also consistent with the master gene model of SINE retroposition which suggests that as a master element accumulates mutations over time, the resulting elements will share those mutations [60].

Members of the *Alu *Ye lineages are dispersed throughout the genomes of all hominoids (humans, greater and lesser apes) suggesting that this subfamily of *Alu *elements began to amplify about 15–20 million years ago. Therefore, the Ye subfamily appears to have been retroposition competent during hominoid evolution, but must have been relatively inefficient at producing copies. Although the rate of Ye amplification has not been dramatic within the human lineage, it may be quite interesting to recover *Alu *Ye subfamily members from other ape genomes and to determine the rate of Ye subfamily amplification in these genomes to see if there has been any differential amplification of these elements in non-human primate genomes. The differential amplification of ID SINEs within various members of the rodent lineage has been reported previously suggesting that the amplification of SINEs within various genomes is subject to changes [61,62].

Gene conversion between *Alu *repeats has been reported previously [26,63,64]. The gene conversion events involve in three *Alu *Ye subfamily members were quite interesting. In one case (Ye5AH181), the *Alu*-containing locus was involved in full gene conversion event where *Alu *Sg in new world monkeys is replaced by an *Alu *Ye5 in Humans, chimpanzees, gorillas and orangutan. In the other two cases (Ye5AH70 and Ye5AH173), only a small portion of the 3' end of the Ye elements were involved in the gene conversion. This is in good agreement with the molecular nature of gene conversion events recently reported for the Ya5 and Yb8/9 *Alu *subfamilies [47,48,64,65]. The detection of three gene conversion events from about 153 *Alu *Ye elements suggests that gene conversion of these events has been relatively rare, with a rate of 1.96%. However, this rate is comparable to that reported previously for the *Alu *Ya5 and Yb8 subfamilies within the human genome, as well as that for the Ta subfamily of human LINE elements [64-66].

In all cases, the Ye *Alu *family members that were involved in the gene conversion were monomorphic for insertion presence within the human genome. In the partial gene conversion events, the Ye *Alu *repeats were gene converted by Yb8/9 and Sx *Alu *elements. The Yb8/9 *Alu *subfamily was one of the first groups of *Alu *repeats that was ever reported to be involved in gene conversion, and may be more prone to these types of events as a result of a retroposition rate that is slightly higher than other recently integrated *Alu *subfamilies in the human genome [48,64,65]. The gene conversion between *Alu *elements may in part be a function of the length of time that the individual *Alu *elements have resided in the human genome [26,50]. Based on an examination of low copy number transgenes in the mouse, it has been suggested that the germline recombination machinery in mammals has been evolved to prevent high levels of ectopic recombination between repetitive sequences [67]. It is quite possible that the high copy number of *Alu *elements allows for pairing between regions of sequence identity of different *Alu *elements initiating the start of gene conversion before cellular control systems can terminate the process resulting in the production of small gene conversion tracts.

The identification of multiple paralogous *Alu *insertions involving an *Alu *Ye element (Ye5AH161) in humans, bonobo, common chimpanzee and gorilla lineage, *Alu *Sp in old world monkeys lineage and *Alu *Sx in new world monkeys lineage is also interesting. The paralogous insertion of an *Alu *repeat into the orthologous regions of human and non-human primate genomes is an independent evolutionary event [26]. To date there are no known cases of the independent insertion of paralogous *Alu *elements into identical sites within different genomes. The detection of parallel insertions is a function of the rate of retroposition of *Alu *elements within various primate lineages and the time since the most recent common ancestor [26]. However, this locus (Ye5AH161) supports the idea of hotspots for the integration of *Alu *repeats within primate genomes. Future studies on the integration of different SINE elements in syntenic regions of human and rodent genomes may yield new insight into the molecular nature of hotspots for SINE element integration.

Genomic deletions created upon LINE-1 retrotransposition using cell culture assays have been recently identified [59]. The rate of LINE element deletion was estimated indirectly in the human genome to be about 3% [68] or 8–13% through sequencing variable sizes of the preintegration sites of L1HS in primates [69]. The precise molecular mechanism of the LINE mediated genomic deletions is still unclear. Recently, an *Alu*-mediated deletion that resulted in the inactivation of the human CMP-N-acetylneuraminic acid hydroxylase gene [70] and *Alu *mediated deletions of noncoding genomic sequences have been identified [71]. Here we report two new examples of *Alu *retroposition-mediated deletions that may have happened by a mechanism similar to that of the LINE element mediated genomic deletions since *Alu *and L1 elements utilize a common mobilization pathway [6,8,72]. In both cases, *Alu *Ye5AH24 and *Alu *Ye5AH27, the deletion appears to have occurred, after the separation of human, chimpanzee and gorillas from orangutan and Siamang, during the process of gene conversion similar to the lineage specific *Alu *deletion reported previously [70,71].

Here, we have estimated the frequency of *Alu *retroposition associated genomic deletions as approximately 1.67%. The size of the deleted sequences was over 300 bp on average. New *Alu *integrations have been estimated to occur *in vivo *at a frequency of one new event in every 10 to 200 births [12]. If sizable deletions accompany one in every 100 new *Alu *retroposition events *in vivo*, the genomic impact of these events could be substantial. This is not a trivial number of deletions when extrapolated to the copy number of *Alu *elements in the human genome which is over one million [2]. Approximately about 16,700 *Alu *elements may have been involved in retroposition mediated deletion events within primate genomes. If each of these deletion events removes an average of 300 bp of genomic sequence, this would mean that *Alu *retroposition mediates the deletion of about 5 Mb of the primate genomic sequences. However, if the *Alu *associated deletions have involved larger sequences similar to those recently reported for LINE elements [59], then the impact of these events may be 50–500 Mb of lineage specific deletions. In either case, these types of events represent a novel mechanism of lineage-specific deletion within the primate order. Detailed studies of the orthologous regions of primate genomes deleted in this manner may prove instructive for understanding the genetic basis of the difference between humans and non-human primates.

## Conlcusion

The Alu Ye lineage has had an extended history of expansion in the human lineage. Its expansion appears to have begun soon after the divergence of the hominoids from the remainder of the catarrhine primates and proceeded at a relatively low level since then. Extended periods of relatively low levels of retrotransposition may allow some mobile elements to retain duplication capability for long periods of time. Despite a relatively low level of retrotransposition, the Alu Ye lineage has contributed to the architecture of the human genome through insertion mutations, retrotransposition associated genomic deletions, and gene conversion.

## Methods

### Computational analysis

To identify *Alu *Ye elements in the draft sequence of the human genome (August 6, 2001, UCSC GoldenPath assembly), we used Basic Local Alignment Search Tool (BLAST) [54] queries of the draft sequence to identify exact complements to the oligonucleotide 5'- GAACCCCGGGGGGCGGAGCCTGCAG-3' that is diagnostic for the Ye lineage as shown in Fig. 1. All of the exact complements to the oligonucleotide queries along with 1000 bp of adjacent flanking unique DNA sequence were excised and stored as unique files and subjected to additional analysis as outlined previously [47-49]. A complete list of all the *Alu *elements identified in the searches is located in Additional file 2 and is available at .

### DNA samples and PCR amplification

Oligonucleotide primers and PCR amplification reactions for each of the *Alu *Ye lineage loci analyzed were performed as previously described [47-49] using the primers and annealing temperatures shown in Additional file 2 for *Alu *Ye lineage members. Diverse human DNA samples were available from previous studies [47-49]. The cell lines used to isolate DNA samples were as follows: chimpanzee (*Pan troglodytes*), WES (ATCC CRL1609); gorilla (*Gorilla gorilla*) lowland gorilla Coriell AG05251B, Ggo-1 (primary gorilla fibroblasts) provided by Dr. Stephen J. O'Brien, National Cancer Institute, Frederick, MD, USA; bonobo (*Pan paniscus*) Coriell AG05253A; orangutan (*Pongo pygmaeus*) ATCC CRL6301; green monkey (*Chlorocebus aethiops*) ATCC CCL70 (old world monkey); and owl monkey (*Aotus trivirgatus*) OMK (OMKidney) ATCC CRL 1556 (new world monkey). Cell lines were maintained as directed by the source and DNA isolations were performed using Wizard genomic DNA purification (Promega). DNA samples from peripheral lymphocytes or tissue were prepared from the gibbon (*Hylobates lar*) and siamang (*Hylobates syndactylus*). Additional non-human primate DNA samples (*Pan troglodytes, Pan paniscus, Gorilla gorilla, Pongo pygmaeus, Macaca mulatta *(old world monkey), *Macaca nemestrina *(old world monkey), *Saquinus labiatus *(new world monkey), *Lagothrix lagotricha *(new world monkey), *Ateles geoffroyi *(new world monkey) and *Lemur catta *(prosimian) available as a primate phylogenetic panel (PRP00001) were purchased from the Coriell Institute for Medical Research.

### Sequence analysis

DNA sequencing was performed on a gel purified PCR products that had been cloned using the TOPO TA cloning vector (Invitrogen) using chain termination sequencing [73] on an Applied Biosystems 3100 automated DNA sequencer. The sequence of the orthologous loci (that contained a paralogous *Alu *element) has been assigned accession numbers AY849282-AY849301. Sequence alignments of the Ye lineage subfamily members were performed using MegAlign software (DNAStar version 3.1.7 for Windows 3.2). The ages for each of the *Alu *Ye subfamilies were calculated using mutation densities as previously described [43,47-49,65] with rates suggested by Xing et al. [56].

## Authors' contributions

AS performed all experimental work for the project, shared in the analysis and interpretation of the results and wrote the first draft of the manuscript. DAR provided assistance with analysis and interpretation of the data and in preparing the manuscript for submission. DJH wrote the software used to extract Ye elements and the associated flanking sequences from the human genome draft sequence. JJ provided assistance with the analysis and interpretation of the data and input on late drafts of the manuscript. MAB provided the initial input for the project as well as valuable input on each draft of the manuscript.

## Supplementary Material

Additional File 1 This supplemental file represents a sequence alignment for for locus Ye5AH161 in fasta format. Click here for file

Additional File 2 This supplemental table lists all Alu Ye elements recovered with information on PCR conditions, chromosomal location and phylogenetic origin. It is in Microsoft Word format. Click here for file
