# Supplementary material for: Analysis of the human Alu Ye lineage
Source: BMC Evol Biol. 2005 Feb 22;5:18. doi: 10.1186/1471-2148-5-18 (PMC554112; doi:10.1186/1471-2148-5-18)
Supplement: Additional File 2 — This supplemental table lists all Alu Ye elements recovered with information on PCR conditions, chromosomal location and phylogenetic origin. It is in Microsoft Word format. [file 1471-2148-5-18-S2.doc]

Supplemental Table. *Alu* Ye elements, Genbank numbers, PCR primers, annealing temperature, primate diversity, location and amplicon sizes.

|  |  |  |  |  | **Primate** ^2^ | **Chr.** | **Product Size** | |
| --- | --- | --- | --- | --- | --- | --- | --- | --- |
| **Name** | **Accession** | **5' Primer sequence (5'-3')** | **3' Primer sequence (5'-3')** | **A.T.^1^** | **Diversity** | **Loc.** | **Filled** | **Empty** |
| **Ye5AH1** | AC109993.3 | AAGTACACCCTGTGATGTTTG | TGCTTGATTCTTCCCTTTTA | **55** | **Gorilla** | **X** | **495** | **242** |
| **Ye5AH2** | AC010205.5 | ACTTCTTCCTGAATGAGTGC | AATAAGCAACCAAACGGAAT | **55** | **Orangutan** | **12** | **497** | **182** |
| **Ye5AH4** | AC117394.10 | GGATTGCTTTTAGTCATCCA | CCCATTCATTAGCTGTTTTC | **55** | **Chimpanzee?** | **3** | **559** | **242** |
| **Ye5AH5** | AC103830.2 | CTCTGACTACTGGGGTTGAA | ATAAAATTTCCGTTCCCTTG | **55** | **Orangutan?** | **8** | **834** | **528** |
| **Ye5AH6** | AC122938.3 | GCTTAATCACTATCTCCATTGTTC | CTGATGTCAATGTTAGCTTTTC | **53** | **Orangutan?** | **4** | **471** | **145** |
| **Ye5AH8** | AC009711.10 | CAGAACCAGGACTCTTTGAG | GGAGTGTGCCTTATGAATGT | **55** | **Chimpanzee** | **15** | **813** | **179** |
| **Ye5AH10** | AC008500.7 | ATCTGTTCAAGGCAGAGAAA | GGACAATTAGTGCCACATCT | **55** | **Gorilla** | **5** | **484** | **184** |
| **Ye5AH11** | AC087297.13 | GTTTTCTCTGTGGGAAGGAT | TTTACCCTTAATGGAGCTTG | **55** | **NA** | **17** | **723** | **408** |
| **Ye5AH12** | AC026325.16 | TTTTAATTCCCAATCAGTGC | GCCCAACATAAACAAATAGC | **56** | **Gorilla** | **3** | **433** | **159** |
| **Ye5AH13** | AC121336.3 | GTTATTTTGAAGGCTTGCTG | TACGGGCTTACAGTGAATTT | **55** | **Orangutan?** | **12** | **432** | **134** |
| **Ye5AH14** | AC022909.13 | TTCAGGAACTGTGAGATTGA | TAGGTGGTAGGCTTTGTGTC | **55** | **Gorilla** | **8** | **500** | **200** |
| **Ye5AH16** | AC007823.38 | GAAAATTAAGCAGCCAGAGA | TAGGGACAAAGAGGATCTGT | **55** | **Orangutan?** | **3** | **498** | **171** |
| **Ye5AH17** | AC108730.7 | GTATCTATTTGGAGCTCTCAGG | CTTCCCTGCCTAGCTCTATT | **55** | **Siamang** | **3** | **494** | **175** |
| **Ye5AH18** | AC106037.9 | TTAGGTCTGTATGGATGAGG | CTGCTCTAGCTCCAAATTGT | **55** | **Siamang?** | **18** | **750** | **442** |
| **Ye5AH20** | AC104838.2 | GAAATGATTCCAGGTTGAGA | CCGGATGGAAGATAATGTAA | **56** | **Human** | **1** | **584** | **296** |
| **Ye5AH21** | AC025458.5 | ATGGAGGAAGGGTAGACAAT | CGTACAGGTCCTTTTCTTTG | **55** | **Gorilla** | **5** | **424** | **110** |
| **Ye5AH22** | AC069218.13 | CCTGACAGGAGGTAGACAGT | CCAAGTATGAGAGTGGAGGA | **55** | **Orangutan** | **3** | **486** | **110** |
| **Ye5AH24** | AC111006.5 | TTCTCTTCTTTCCCAACTGA | GCCTAGCCAAGTTGACAATA | **55** | **Gorilla** | **4** | **440** | **148** |
| **Ye5AH27** | AC006255.9 | ACACTACCTAAAACTGCCCAGAAG | ACTATCTCACCCTCCAAAAACACT | **60** | **Gorilla** | **3** | **476** | **161** |
| **Ye5AH29** | AC011939.9 | CCTCTCATTACATCTTTCATAGC | TGGAATGTATTATCTAGAGCACAG | **55** | **Orangutan** | **15** | **485** | **170** |
| **Ye5AH30** | AC009097.8 | CATGAATTAAATACCCACTGC | CCCTCATGGAACACTTTTAG | **55** | **Orangutan?** | **16** | **696** | **380** |
| **Ye5AH31** | AC111149.4 | AATGGAGGAGGAGATAGGAA | AGCTCTGTTGGCAGAATTAG | **60** | **Chimpanzee** | **8** | **840** | **364** |
| **Ye5AH32** | AC104006.6 | ATTGCCGAGAGTAGATTTCA | TTACCTTCCCTAACCACTACC | **60** | **Orangutan?** | **8** | **697** | **382** |
| **Ye5AH34** | AC092851.9 | TTGAGCAAATCACTCACCTTC | CAGTGAAAGGGGAGGAAAAT | **55** | **Orangutan?** | **12** | **481** | **178** |
| **Ye5AH35** | AC106752.3 | GAGTTGCTATGACAATTGGAG | GTTTCAGCTCTTGTGGATTC | **55** | **Gorilla** | **5** | **467** | **149** |
| **Ye5AH36** | AC108016.5 | CAAAACATTTGGCCTATTTC | AGAATGGTGGATTTTGAATG | **55** | **Orangutan** | **4** | **387** | **277** |
| **Ye5AH37** | AC106872.5 | CTCTTAACATGGGCTACCTG | GCAATGAAGAACTCTGTGGT | **60** | **Gorilla** | **4** | **477** | **169** |
| **Ye5AH38** | AC093516.4 | GCCATCTGTGGTCAAGTAAG | CCATGAGATTGTCTCTGGTT | **55** | **Orangutan** | **16** | **797** | **158** |
| **Ye5AH39** | AC024575.6 | TGAGGGAAGAGAAAAACTGA | GTGGTCTGGCTTCTAGAGTG | **55** | **Siamang** | **19** | **995** | **696** |
| **Ye5AH40** | AC090179.4 | TGTACTACACGCAGTCTTCC | CGTGGGTGTTATCTTAGCTC | **60** | **NA** | **15** | **975** | **139** |
| **Ye5AH41** | AC099518.2 | GTGCTCATGATGCTCACATA | AGTGATGAACCTCGACTCAG | **60** | **Orangutan?** | **16** | **995** | **671** |
| **Ye5AH43** | AC093557.3 | AACTGGAGCATTTTACTGAGA | AATGTGCCCATATCTGAAAG | **55** | **Gorilla** | **3** | **574** | **154** |
| **Ye5AH44** | AC097720.5 | AGAGATATGGCATCCCTTTA | GGAAGATATTATTGGGCAAA | **55** | **Orangutan** | **2** | **489** | **178** |
| **Ye5AH45** | AC109811.4 | GTCAAGATGTGAAGGGCTTGTAGT | TATTGCCAGTTTGCACTAGTCATT | **60** | **Siamang?** | **4** | **499** | **179** |
| **Ye5AH47** | AC012493.9 | CATTATGAAGGTTCCTGCTC | ATGGGAAACTAACCAAACCT | **60** | **Orangutan?** | **2** | **373** | **258** |
| **Ye5AH48** | AC013459.9 | GGTGGCCACTTTCCTATATT | AGAGACCAAAGTTCAACCAA | **60** | **Gorilla** | **2** | **394** | **~94** |
| **Ye5AH50** | AC091534.7 | TTCAAACTCCTGCCGTCTTT | GGCTGAGATCCTGGTTCAGA | **60** | **Orangutan?** | **12** | **525** | **109** |
| **Ye5AH52** | AC097516.2 | TGCTTCTGAGTCCTAGTTCC | CCTGTTTGTCCAATAACCTT | **60** | **Siamang** | **4** | **373** | **261** |
| **Ye5AH53** | AC097511.1 | TGGGCAATCCAAAATGAAAT | TAGGAATTGCATTGCACCAG | **60** | **Orangutan?** | **4** | **546** | **230** |
| **Ye5AH54** | AC096562.1 | TTGTTTTCATTGCTTGTACTCTTC | GAAAATTCTTCTGGACATTTGGT | **55** | **Orangutan** | **7** | **396** | **75** |
| **Ye5AH55** | AC096661.1 | CCAGTTTCATGAAGTGAACA | ATGGGTTGTCAATCTGATGT | **55** | **Orangutan?** | **4** | **477** | **180** |
| **Ye5AH56** | AC098582.2 | GTGAAGAGAACAAGGTGAGC | CATTTTAATGGCAGCTAAGG | **55** | **Siamang** | **4** | **442** | **122** |
| **Ye5AH57** | AC093724.2 | CCTGTACCTTGAAACTACACATGG | AGTGCCACTCATGTGGTATCTAAT | **60** | **Siamang** | **2** | **295** | **138** |
| **Ye5AH59** | AC097530.3 | TTTCTATGTCTTTCTCCATGCTCA | CAGAATAAGGAATGATAACGGAATG | **60** | **NA** | **4** | **850** | **539** |
| **Ye5AH60** | AC022960.13 | GTTGTGAACACAGGAGAGGT | AGTAAACAGCTTTGCAGCTT | **60** | **Siamang** | **18** | **828** | **520** |
| **Ye5AH61** | AC013746.10 | GCATGTGTCTGAGCAAGAGC | TCAGGGCCCTTTTAACTGTG | **60** | **Gorilla** | **8** | **478** | **257** |
| **Ye5AH63** | AC069413.14 | AAAGGGGAGGGAAAGCACTA | ACATTGATGGTGGTTTGCTG | **63** | **Orangutan** | **3** | **408** | **96** |
| **Ye5AH64** | AC008885.5 | TTCCTACCCAATTATGAACG | CCACTTTATCTCACACATTCC | **55** | **Orangutan** | **5** | **416** | **103** |
| **Ye5AH66** | AC073284.5 | CAGGCCCCAGAGTAAACATC | TGAGCCTTTTCTGCTTGGAT | **60** | **Gorilla** | **2** | **846** | **~215** |
| **Ye5AH68** | AC012510.8 | CCAGAGGCTATTGATGTAGC | CATTTTGTCTACTCCCTTGC | **55** | **Siamang** | **2** | **465** | **159** |
| **Ye5AH69** | AC068721.4 | TGGGTAGTGATGTTTGGACATTAT | TCAGTTTAATTGAAGGGACATTCTC | **55** | **Orangutan** | **4** | **489** | **156** |
| **Ye5AH70** | AC017084.8 | GAGTTAATGATGCAAAAAGGTGCT | CATCTTAGTGTATGTCCTTCCCAGA | **55** | **Gorilla** | **2** | **450** | **129** |
| **Ye5AH71** | AC066692.3 | AGGGGGAAAAATAGGCATCA | TGCTTTCGCAGACCCTTATT | **60** | **Orangutan?** | **2** | **456** | **145** |
| **Ye5AH73** | AC015969.9 | AAATAGCCAGTTATCCCAGAATCA | TCTCATTTATGATGACCTTCTCCA | **60** | **Orangutan** | **2** | **577** | **270** |
| **Ye5AH75** | AC009126.2 | AGTCAGATGACTCCCAGACA | GGCCACTATTGGTGCTATAA | **60** | **Orangutan** | **5** | **738** | **108** |
| **Ye5AH76** | AC093263.3 | CGCTGTTGTTCTTTGTAGTTT | CAGTATTTGGAAACAAAGCA | **60** | **Orangutan?** | **5** | **439** | **120** |
| **Ye5AH77** | AC092080.2 | CACCTGGGCCAAAAGATAAT | GACAACAATGCAGCAAATGG | **60** | **Siamang** | **19** | **581** | **260** |
| **Ye5AH80** | AC092811.2 | TTTATGCGTGGATGTCTAAGTTGT | GCAGTAATCAAGACTGTGTGATCC | **55** | **Orangutan** | **1** | **483** | **174** |
| **Ye5AH85** | AC008916.7 | CAGGCAGTGTATTCATTCAA | GTTCTGCTCCTTTTCCCTAT | **55** | **Human** | **5** | **459** | **141** |
| **Ye5AH86** | AC093218.2 | GGCAAAGCAGTTTCAGTAAT | GGTTCATTTAGGTTTGCATC | **60** | **Gorilla** | **5** | **598** | **297** |
| **Ye5AH88** | AC010457.7 | CTCTTCCTGTCGTCAGAGTC | ACAATGAGAAGCCAATGAAG |  | **No PCR** | **5** | **444** | **117** |
| **Ye5AH89** | AC016465.8 | TTTCTCTCTCTCTCTTTCCCTCTC | CTCTTGAAACCAATGAAGAAGGTT | **55** | **Human** | **8** | **460** | **152** |
| **Ye5AH92** | AC010425.6 | TTCAAAGTGAATGGACAACA | CTTCCACCACTGTTCAATTT | **55** | **Siamang** | **5** | **448** | **134** |
| **Ye5AH93** | AC027139.5 | ACAAAGAGATACGCGCATACATAA | ACTCCATGACTCTTCCTTTCACAT | **60** | **Chimpanzee** | **15** | **423** | **109** |
| **Ye5AH95** | AC007991.7 | TGTTCCTCAATACCATAAAGG | AGACGAAAGCATTCAACTGT | **55** | **Siamang** | **8** | **401** | **84** |
| **Ye5AH97** | AC008171.3 | TGTTTATTGGAGGTAAGAGGAAGC | GTGATGGAAGCATTCGTATAACTG | **60** | **Gorilla** | **2** | **471** | **151** |
| **Ye5AH99** | AC083872.2 | CCTTTAGAAATGAAGACCCAAAGA | CAGTGTATTACCATTAGATGGTTGC | **55** | **Orangutan** | **7** | **589** | **90** |
| **Ye5AH100** | AC068716.8 | GGTTCCTGGGGGATTATAAACTAT | AAATTCAAACCACCACCCTAAAC | **55** | **Siamang** | **15** | **403** | **108** |
| **Ye5AH102** | AC083863.2 | ATGCCATTTTATCCACAACT | CACCCAGAAATTTGGTAACT | **60** | **Gorilla** | **7** | **433** | **117** |
| **Ye5AH103** | AC009478.4 | CCCCAGTCCCCTATAGAAGATAAT | TCAAACAAACTGGGATACTTCTGA | **60** | **Orangutan** | **2** | **464** | **313** |
| **Ye5AH104** | AC011890.4 | TACCTGTACCCGTATTTCATTCCT | TGTTCTAGTGGTATTGTGGGTTTTT | **55** | **Chimpanzee?** | **X** | **454** | **151** |
| **Ye5AH109** | AC008102.17 | TGCCAGACACATCATCTAAG | CATTCCTGGAAAATTTAGTG | **55** | **Gorilla** | **11** | **425** | **122** |
| **Ye5AH110** | AC009517.5 | GTATAAAGGGATGAAACAAGCTGAA | GTGTATTCTTGTGTGGTATTGTTTC | **60** | **Human** | **7** | **500** | **185** |
| **Ye5AH112** | AC007957.36 | ATTTGAAGGAACGACTGAAA | GGTGATTTATGTGCTTCCAA | **55** | **Orangutan** | **22** | **496** | **182** |
| **Ye5AH114** | AL390865.10 | TCCCCACATGCTATGACAGA | TGCTGGTGAATGAATGCATATAG | **55** | **Human** | **10** | **500** | **187** |
| **Ye5AH115** | AL158040.14 | CCTCAGGATTCTACTACTGGTT | GTTCAACTCTTTGCCACATC | **55** | **Chimpanzee** | **10** | **684** | **375** |
| **Ye5AH116** | AC006063.1 | TTCTCTCCCTGCTCCTTCTG | TGAAGGGAGAATTTGTGAACC | **55** | **Siamang** | **12** | **445** | **125** |
| **Ye5AH117** | AL132639.4 | CGTGTTTCCCCTCAATGAAT | CACCCAGCTTGTGTAACATTT |  | **No PCR** | **14** | **834** | **467** |
| **Ye5AH119** | AL109917.22 | AGGTATGCAAAGAACAAAGC | CAAAGGTTGCTGGATTTAAC | **55** | **Gorilla** | **1** | **828** | **523** |
| **Ye5AH120** | AC016830.5 | CTTTGGCAGAATCTTGGAATG | TCTTAAAGGATGATGGGAGTTTGT | **55** | **Gorilla** | **22** | **537** | **221** |
| **Ye5AH122** | AC005409.1 | TGGTCTCATCAGGGGAAAAG | AGCTCTCTGGGATGTTGTGC | **60** | **Gorilla?** | **10** | **443** | **128** |
| **Ye5AH123** | AL590714.27 | GCTCATGAATGAGTTCTTCC | TGCTCACATGAGACAAGAAA | **55** | **Siamang** | **1** | **542** | **406** |
| **Ye5AH124** | AL358472.54 | CTACACTCCTCCGATACCAG | TAAGAGGCCAGTCTTGACAT | **55** | **Siamang?** | **1** | **457** | **141** |
| **Ye5AH125** | Z94044.2 | TAAAATCATGAGAAACTGGGACTG | GTTAAATTTGCTCTTCCCACGTAT | **55** | **Gorilla** | **X** | **478** | **162** |
| **Ye5AH126** | AL359259.18 | TGGGCATAGGAGTACAAACT | ACGGTGAACACAAAAGATTC | **55** | **Gorilla** | **1** | **486** | **174** |
| **Ye5AH128** | AL592301.14 | CAATGCAACTGCCTATCACC | AAGATCAGCTGGCCTCTTTG | **55** | **Gorilla** | **9** | **425** | **102** |
| **Ye5AH131** | AL355872.15 | CTCTTGACTGACTTAGTAACTTCAT | TGCTCCAACATTACAATGAG | **55** | **Gorilla?** | **9** | **395** | **85** |
| **Ye5AH134** | AL080238.10 | GGTGCATTCTTGGTGGTACTAAT | ATCACTTTAGTTGGAGTCCCTCTG | **55** | **Gorilla** | **X** | **458** | **155** |
| **Ye5AH137** | AL590812.8 | CATGCCTACCTTCAACATTT | GGACATTGGGTGTTCTTCTA | **55** | **Gorilla?** | **9** | **600** | **287** |
| **Ye5AH138** | AL513315.15 | TGACATAGGAGAAGGAAACAA | CCACCCTTCTCATTTACAGA | **54** | **Gorilla?** | **1** | **579** | **245** |
| **Ye5AH139** | AL513302.15 | TGAGAGAGACATATCCTATTTCTGGA | TTTCTTGGCTATTCTCATCTGTCA | **55** | **Orangutan?** | **1** | **489** | **188** |
| **Ye5AH140** | AL139156.11 | GTGAATGCAGATCATTATGGAAAG | GGGAAAAGATGCAGTGTAGAGTTT | **55** | **Gorilla** | **1** | **507** | **183** |
| **Ye5AH141** | AL590632.5 | CATCCCTGCATAGTTTTCATTG | ACCATCTAAAATCCAACGGAGA | **60** | **Gorilla** | **1** | **911** | **582** |
| **Ye5AH142** | AL353689.26 | TCATCCAAACATCTCAATGC | CAAATCATAACTTGCTAGATACTCC | **55** | **Gorilla?** | **1** | **500** | **182** |
| **Ye5AH143** | AL353671.6 | CACTAAAGGTGAATCGTTGC | GGACACATTTTAATGCCACT | **55** | **Siamang?** | **9** | **440** | **94** |
| **Ye5AH144** | AL157936.12 | TTCCACCACAATTTAAGGTC | TCAAAAGGTCACCTGTCTTC | **60** | **Orangutan** | **9** | **495** | **188** |
| **Ye5AH145** | AL390859.18 | GTGGCCTTAAAGCTGAGTAG | CTTGTAGACATTATCATCTGAAGC | **55** | **Gorilla** | **10** | **489** | **163** |
| **Ye5AH146** | AL139099.3 | ATTACTTGGGCAGATGGTTA | CAAGATAAGCAATATGGAAGC | **55** | **Gorilla** | **14** | **489** | **180** |
| **Ye5AH147** | AL161786.12 | GCTGTAATAAACACCCTTGC | ACTGATAAAGCCACTTTGGA | **55** | **Siamang** | **9** | **463** | **152** |
| **Ye5AH148** | AC010072.5 | TAGATAGCAGCCCCATTCCA | CCTTTCGACACGGGAAAGTA | **55** | **Siamang** | **14** | **461** | **126** |
| **Ye5AH150** | AC002476.1 | CCAAAGTGAAAATTGGTCAT | CCAAGACCAGAGACAGGTAG | **55** | **Siamang?** | **X** | **483** | **181** |
| **Ye5AH151** | AC003084.1 | TAAATGTTTCCCTGGATAACCCTA | GAAAACGAAGTGCTCATTCAGTAA | **60** | **Gorilla** | **7** | **470** | **154** |
| **Ye5AH153** | AL513008.14 | TACATTTCAGCAGTCCCTTC | TAGGATGAGCATTTTCCAAT | **55** | **Orangutan** | **6** | **807** | **483** |
| **Ye5AH154** | AL157955.5 | GTTTTGTCCTTTCTGAATGC | CATTGAGGAAACCTCTTCAG | **55** | **Chimpanzee?** | **14** | **432** | **115** |
| **Ye5AH155** | AL512802.2 | TAAAGCATATGACAGGATGG | TCCATTTTGGTTCCACTATT | **55** | **Siamang?** | **14** | **500** | **193** |
| **Ye5AH156** | AL121767.6 | AATGACAGACCCGGAGACAC | GACACAGGACCCTTGGAGAA | **55** | **Orangutan?** | **14** | **433** | **108** |
| **Ye5AH157** | AL079306.3 | CAGGAAAGGGGACAGGAAGT | AAATCCATTCAGGGCATCTG | **55** | **Orangutan?** | **14** | **491** | **180** |
| **Ye5AH160** | AL450344.4 | TGTTGGTTGCATGGAGACAT | GATGCAAGCCTGTGATGAAA | **60** | **Human** | **6** | **483** | **236** |
| **Ye5AH161** | AL365364.19 | CACAGAATTACTTCCCCTTTATGG | ACCTGTTATATGGTTTGCCCTCT | **60** | **Gorilla** | **10** | **795** | **486** |
| **Ye5AH162** | AL359081.10 | TTCCCTCTGAATGTGTGCAG | GGCTACTCTGCCCCCACTAT | **55** | **Siamang?** | **1** | **449** | **132** |
| **Ye5AH165** | AL157768.6 | CCTTCTTTTTGCCTATTGTG | AAAGGAGGCATTATTTTTCC | **55** | **Siamang?** | **13** | **795** | **485** |
| **Ye5AH166** | AL133480.9 | TTCCATCCTGATTCCTTCTA | AAACAGAAGCGTAAACCAAA | **55** | **Gorilla** | **9** | **537** | **209** |
| **Ye5AH167** | AC004067.1 | AGCCTGTCACCTGACTTAAT | ACTGAGCCAAACTCTTTTGA | **55** | **Human** | **4** | **593** | **268** |
| **Ye5AH168** | AL121715.15 | TGTCTTATCAAACCCCCTTA | GCATAACTTGGACAAAGAGC | **55** | **Orangutan?** | **6** | **594** | **301** |
| **Ye5AH169** | AL450270.7 | GGATTGTCCTCAGTGTATGTTGAA | CTTCGGAATGATCTTTCTTCACAG | **61** | **Orangutan?** | **6** | **499** | **185** |
| **Ye5AH170** | AL135749.3 | CCATCTCCTTATTGATGGAT | TGTTTGCTGCATAAATCTCA | **55** | **Chimpanzee** | **X** | **451** | **147** |
| **Ye5AH172** | AL138958.18 | ACATTTCGCTTGTCCCTTTC | CAAAGAAACGGACGTTGCAC | **55** | **Siamang** | **13** | **496** | **192** |
| **Ye5AH173** | AL136304.10 | GCACGAGCCTGTGAGAGTG | AGGGAAGATATCCCTGAAGAGG | **55** | **NA** | **6** | **436** | **697** |
| **Ye5AH175** | AC004559.1 | TTTTCTTTCGCAGTGTTCTT | GAGGTCTGCATTCACAGACT | **59** | **Gorilla** | **19** | **795** | **477** |
| **Ye5AH176** | AC003664.1 | TTCACCAAGAAGATATACGACTGG | AATGTTCCTGTGTCTCCATATCCT | **60** | **Gorilla?** | **17** | **494** | **182** |
| **Ye5AH177** | AL033543.6 | GGGCTGTACAACTGTGTTTT | TCAACGTTCTTCTGTTTGAA | **58.8** | **Human?** | **22** | **850** | **510** |
| **Ye5AH181** | AP000432.4 | TTTATCTGCTCTTTGGAAGG | TGATTGCTAACCTCCAATTT | **54** | **Orangutan?** | **21** | **468** | **158** |
| **Ye5AH190** | AP001201.7 | CCAAGGGGCTCTGTCTACAA | GCATGAATTCCTCAGGCTCT | **55** | **Siamang?** | **11** | **446** | **133** |
| **Ye5AH191** | AP003064.2 | TGGAAGGAATTAGGCACCAG | CAGCGACAGGTTCATAGCAA | **55** | **Gorilla** | **11** | **534** | **224** |
| ^1^. Annealing temperature. | | | | | | | | |
| ^2^. Primate diversity is defined as: Human, the insertion only appeared in human genome, not any others. Chimpanzee, the insertion was found in human, bonobo and common chimpanzee genome. Gorilla, the insertion was found in human, bonobo, common chimpanzee and the gorilla genome. Orangutan, the insertion was present in human, bonobo, common chimpanzee, gorilla and orangutan genome. Siamang, the insertion was present in human, bonobo, common chimpanzee, gorilla, orangutan, siamang genome. A question mark indicates that a filled site amplified from the species listed and an empty site did not amplify in the next taxon in the panel. NA indicates no amplification. | | | | | | | | |
